# Bone Mineral Density Measurements, Bone Markers and Serum Vitamin D Concentrations in Men with Chronic Non-Cirrhotic Untreated Hepatitis C

**DOI:** 10.1371/journal.pone.0081652

**Published:** 2013-11-28

**Authors:** Luciana G. S. Orsini, Marcelo M. Pinheiro, Charlles H. M. Castro, Antônio E. B. Silva, Vera L. Szejnfeld

**Affiliations:** 1 Rheumatology Division, Universidade Federal de São Paulo/Escola Paulista de Medicina (Unifesp/EPM), São Paulo, Brazil; 2 Gastroenterology Division, Universidade Federal de São Paulo/Escola Paulista de Medicina (Unifesp/EPM), São Paulo, Brazil; INSERM U1059/LBTO, Université Jean Monnet, France

## Abstract

**Introduction:**

The high prevalence of chronic hepatitis C (CHC) and its consequent cirrhosis has been associated with bone fragility. Whether CHC may cause bone and mineral abnormalities in the absence of hepatocellular dysfunction is still unknown. In this study we aimed to determine the prevalence of osteoporotic vertebral fractures and low BMD measurements in men with non-cirrhotic CHC. Risk factors for low BMD and fractures were also investigated.

**Methods:**

Morphometric vertebral fractures and BMD measurements were performed in 60 non-cirrhotic untreated men with CHC and 59 healthy controls, matched for age and gender, weight and current smoking. Serum CTx, calcium, phosphate, intact PTH, alkaline phosphatase and vitamin D (25OHD) concentrations were measured in all participants. Clinical risk factors for low BMD and fractures were evaluated by a structured questionnaire as well as details regarding HCV infection.

**Results:**

Trochanter and total femur BMD were significantly lower in CHC patients as compared to healthy men (p = 0.04). In men 50 years and older, the prevalence of osteoporosis was significantly higher among CHC patients (p = 0.01). Lower levels of physical activities and more often report of prolonged immobilization were observed among CHC patients (p<0.05). Liver inflammation and fibrosis, viral load and genotype did not correlate with BMD measurements. Bone markers and 25OHD concentrations were similar in both groups. Only a few vertebral fractures were observed.

**Conclusions:**

Our results demonstrate that non-cirrhotic untreated CHC patients have lower BMD at the femur as compared to healthy men in spite of the absence of significant bone and mineral abnormalities.

## Introduction

The World Health Organization (WHO) has estimated that about 3% of the world population has positive serology for hepatitis C virus (HCV) (approximately 170 million of people) [1]. Bone diseases are common complications of chronic hepatitis C (CHC), mainly related to the associated cirrhosis. Among the bone abnormalities reported, hepatitis C-associated osteosclerosis (HCAO) is a rare acquired condition characterized by generalized increase in bone mineral density (BMD), bone pain, elevated serum alkaline phosphatase, generalized cortical thickening and increased uptake on bone scan [2]–[4].

Bone loss has been described as a complication of viral cirrhosis [5]–[9] and its prevalence increases in more severe cases [5]–[7]. Higher levels of serum soluble tumor necrosis factor (TNF) receptor 55 [8] and lower levels of 25-hydroxy vitamin D (25OHD) [5], [7], [8], testosterone [5], [7] and IGF-I in more advanced Child-Pugh stages [7], [8] may all contribute to the lower BMD measurements observed among patients with more severe disease.

Bone disorders have been clearly associated with the presence of cirrhosis per se, regardless of its cause. In patients with CHC and other viral hepatitis, their treatment may also be considered as a confounder when evaluating bone complications. It has been reported that the combination therapy for patients with CHC consisting of interferon-α (INF-α) plus ribavirin resulted in bone loss after 12 months [10]. Increased BMD as well as no change have also been reported after treatment with INF-α in patients with CHC [11], [12].

On the other hand, BMD and bone metabolism abnormalities in non-cirrhotic hepatitis C patients have not been well investigated. Whether chronic HCV infection in the absence of cirrhosis or viral treatment is a risk factor for the development of bone disease has long been controversial.

Only a few studies have evaluated bone status in CHC patients [13]–[15]. Some methodological questions associated to current treatment in some of the patients and the absence of a healthy control group for comparison has limited the results of those studies. Considering the lack of solid evidence for an effect of chronic HCV infection on bone status, in the present study we evaluated BMD measurements, bone and mineral metabolism and the prevalence of vertebral osteoporotic fractures in non-cirrhotic men with untreated CHC infection as compared to healthy age-matched controls.

## Materials and Methods

### Ethics Statement

Ethical approval was obtained from the Universidade Federal de São Paulo's ethics committee and all participants gave written informed consent prior to their inclusion in this study.

### Patients

Sixty men (mean age 41.5±10.9 years old) with CHC infection consecutively seen at the UNIFESP's outpatients clinics and fifty-nine healthy men (mean age 41.7±10.8 years) were invited to participate in the present study. Anti-HCV/HCV RNA-positive patients (N = 60) had been followed for at least 6 months with persistently positive serology for HCV and negative serological markers for hepatitis B and human immunodeficiency virus (HIV). Healthy men (N = 59) were selected from blood donators registered at our center.

CHC patients with any clinical, laboratorial or histological cirrhosis were excluded, as well as those previously or currently treated with interferon/ribavirin therapy. Other exclusion criteria for both CHC patients and healthy controls included alcoholism (defined as more than 2 drinks per day) [17], diseases or use of drugs that interfere with bone metabolism (corticosteroids, levothyroxine, calcium and vitamin D supplements, diuretics, bisphosphonates, anabolic agents), diabetes mellitus, lung diseases and autoimmune hepatitis.

Body weight was measured (after removal of shoes and heavy outer clothing) using a balance beam scale. Height was measured (after removal of shoes) using a Filizola stadiometer. Height and weight were used to calculate the body mass index (BMI; kg/m^2^). Data about smoking and drinking habits, physical activity, prolonged immobilization, familial history of osteoporosis and hip fracture were obtained using a structured questionnaire based on the European Vertebral Osteoporosis Study (EVOS) [18], [19] and validated for our language and culture [20]–[22]. Patients and controls were matched for age, gender, weight, current smoking and alcohol use.

### Bone density measurements

BMD measurements were performed in all subjects at the lumbar spine (L1-L4) and proximal femur (neck, trochanter and total hip) using dual X-ray absorptiometry (DXA) (DPX MD+, GE-Lunar, Madison, WI, USA). The coefficient of variation for BMD measurements was 1.5% and 2% at lumbar spine and total hip, respectively. Long-term quality control of the instrument was assured by daily scan using a standard spine phantom as instructed by the manufacturer. All scans were performed in the same machine by the same operator and were analyzed by a physician blinded to the group the participant belonged. Consistent with the recommendations from WHO [23] and from the International Society of Clinical Densitometry [16], men 50 years and older were classified according to T-score as normal bone density (T-score above – 1.0 SD), osteopenia (−1.0< T-score <−2.5) or osteoporosis (T-score below – 2.5 SD). Z-score was used to classify younger men (less than 50 years old) as within the expected range for age (Z-score above – 2.0) or below the expected range for age and sex (Z-score below – 2.0). For comparison purposes, BMD measurements in both groups were adjusted for age and body weight.

### Laboratory tests

Blood was drawn in the morning after an overnight fasting. Routine tests included total serum calcium, phosphate, and creatinine and total alkaline phosphatase using standard methods. Intact parathyroid hormone (iPTH) (Nichols Advantage® Chemiluminescence Intact Immunoassay; interassay coefficient of variation of 7%; normal range 10–65 pg/mL), 25-hydroxy vitamin D (25OHD) (radioimmunoassay technique, DiaSorin, Stillwater, MN, USA; intra- and inter-assay coefficients of variation were 9.5% and 15.2%, respectively) and type I collagen C-telopeptide (CTx) serum concentrations (Elecsys 2010 automated analyzer, Roche; interassay coefficient of variation of 6%; normal range 0.081–0.850 ng/mL) were also determined.

HCV viral load and genotype, liver biopsy classification and serum albumin were obtained from an extensive review of the patients' charts.

### Vertebral fracture assessment

Radiographic lateral and posterior-anterior images of thoracic and lumbar spine were taken according to standard protocols aiming to evaluate asymptomatic vertebral fractures. Thoracic films were centered at T8 and lumbar films at L3. Focus film distance was 1.5 m. A semi-quantitative method [24] was used to determine the prevalence of vertebral deformities. Only grades II and III were considered as vertebral fractures. Intra and inter-observer coefficients of variation and kappa indexes for the method in our unit are 4.8% (concordance 95.2%) and 6.3% (concordance 93.7%) and 0.84 (95%CI 0.78–0.92) and 0.82 (95%CI 0.75–0.9), respectively [21].

### Statistical analysis

Results were expressed as mean ± standard deviation. Chi-squared tests were used to evaluate the prevalence of osteoporosis, osteopenia, and low BMD as well as the vitamin D status between CHC men and their healthy controls. We compared the BMD, anthropometric variables, frequency of risk factors for osteoporosis and laboratorial tests between CHC patients and healthy controls using Student's t-tests. Two-way ANOVA analyzes were used to adjust vitamin D concentrations according to the season of the year. Pearson's and Spearman's coefficient of correlation were used to estimate association between clinical variables and BMD measurements with the prevalence of low bone mass and osteoporosis in CHC patients. Potential risk factors for low bone mass and osteoporosis were analyzed by backward stepwise regression analysis using variables identified in correlation analyzes. SPSS statistical package for Windows (version 15.0) was used to analyze the data. Significance level was set as p<0.05.

## Results

Relevant demographic and clinical characteristics for both CHC patients and healthy men are shown in Table 1. CHC patients had lower physical activity and reported prolonged immobilization more often than healthy men (p<0.05). In spite of their similar weight and height, CHC patients had significantly higher BMI values as compared to the healthy controls (p = 0.04).

**Table 1:** Demographic data and clinical characteristics of chronic hepatitis C (CHC) men and healthy controls.

	CHC (N = 60)	Controls (N = 59)	*p* *
Age (years)	41.50±10.90	41.70±10.80	0.92
Weight (kg)	77.50±15.70	79.60±11.70	0.07
Body mass index (kg/m^2^)	26.10±4.60	26.80±3.00	**0.04**
Smoking, n (%)	18.00 (30%)	18.00 (31%)	0.95
Lifetime tobacco exposure (pack-years)	27.00±12	28.00±11	0.71
Drinking habits, n (%)	27.00 (45%)	39.00 (66%)	0.07
Alcohol intake (drinks/week)	3.80±3.40	4.10±3.10	0.53
Physical activity **, n (%)	14.00 (23%)	24.00 (40%)	**0.04**
Previous prolonged immobilization, n (%)	14.00 (23%)	2.00 (3%)	**0.001**
Family history of osteoporosis, n (%)	11.00 (18%)	15.00 (25%)	0.34
Family history of femur fracture, n (%)	5.00 (8%)	9.00 (15%)	0.24

** Physical activity at least 3 times/week, for 30 minutes;

* Student t-test

Spine and femoral neck BMD did not differ significantly between HCV patients and healthy men, as demonstrated in Table 2. On the other hand, total femur and trochanter BMD values were significantly lower in CHC men as compared to healthy controls, even after adjustments for body weight (p<0.05).

**Table 2:** Bone mass measurements in CHC patients and healthy controls.

Bone mineral density (g/cm^2^)	CHC (N = 60)	Controls (N = 59)	p*
Spine (L1–L4)	1.168±0.15	1.165±0.15	0.96
Femoral neck	1.029±0.17	1.061±0.17	0.49
Trochanter	0.870±0.13	0.923±0.13	**0.03**
Total Hip	1.051±0.15	1.111±0.15	**0.04**

*Student t-test

For subjects younger than 50 years old, BMD measurements below the expected range for age and sex (Z-score below – 2.0) were found in 1 (at the femur) out of 41 CHC patients (3%) and 2 (one at the femur, one at the lumbar spine) out of 40 controls (5%). Again, no statistically significant difference was observed between groups. For men 50 years old or more, osteoporosis was detected in 36% of the CHC patients (two patients at both femur and lumbar spine, three patients at the femur and two other patients at the lumbar spine) and in only 4% of the healthy men (2 at the femur and two others at the lumbar spine). Osteopenia was observed in 32% of both groups (three patients at the femur and three others at the lumbar spine; five healthy controls had osteopenia at the lumbar spine and one of them had osteopenia at the femur). BMD classified as normal was found in 32% of the CHC patients and in 64% of the controls (*p* = 0.01).

Serum concentrations of biochemical bone markers were in the normal range for both CHC patients and controls as shown in Table 3. Total calcium, phosphate, alkaline phosphatase, iPTH, CTx and vitamin D did not differ significantly between HCV patients and controls. The mean serum albumin was also within the normal limits in CHC patients (4.25±0.4 mg/dL).

**Table 3:** Biochemical bone markers and laboratory tests of bone and mineral metabolism in CHC men and healthy controls.

	CHC (N = 60)	Controls (N = 59)	*p* *
Total calcium (mg/dL)	9.09±0.40	9.08±0.30	0.76
Phosphate (mg/dL)	3.11±0.40	3.04±0.30	0.32
Creatinine (mg/dL)	0.89±0.10	0.91±0.10	0.41
Alkaline phosphatase (UI/dL)	85.50±68.02	72.70±19.20	0.44
Intact PTH (pg/mL)	56.20±22.04	51.90±23.02	0.19
CTx (ng/mL)	0.40±0.33	0.42±0.15	0.07
25 OHD (ng/mL)	30.60±10.02	28.50±8.10	0.16

*Student t-test

Using previously published criteria [25], 25OHD status was classified in patients and controls as follows: sufficiency (above 30 ng/mL), insufficiency (11–29 ng/mL) and deficiency (below 10 ng/mL). After adjustments for the season of the year, no statistically significant difference was observed between the groups in terms of 25OHD status (Table 4).

**Table 4:** Classification of vitamin D status in CHC men and healthy controls.

25 OHD (ng/mL)	CHC (N = 56)	Controls (N = 59)
Sufficiency (>30)	32 (57%)	18 (31%)
Insufficiency (11–29)	23 (41%)	41 (69%)
Deficiency (<10)	1 (2%)	0

*p* = 0.07 (Chi-squared test); adjusted to the season of the year (ANOVA)

Chi-squared test and linear backward stepwise regression analyses were performed to investigate potential determinants for low BMD in our sample. BMD measurements at all skeletal sites did not correlate with lifetime tobacco exposure (pack-years of smoking), alcohol consumption (drinks/week), physical activity [26], prolonged immobilization, time since diagnosis and contamination, viral load and genotype, degree of liver inflammation and fibrosis at the biopsy in CHC patients. Although CTx serum concentrations correlated negatively with lumbar spine BMD (r = −0.21; *p* = 0.04), no other significant correlation was found between BMD measurements and other biochemical markers. Multivariate analyzes performed did not allow to identify any parameter significantly associated with low BMD measurements in CHC patients.

Morphometric vertebral fractures were observed in three healthy men and one CHC patient. The CHC patient was a 57 years-old man with history of current smoking (40 pack-years) and alcohol use (10 drinks/week). BMD measurements, bone markers and vitamin D status were in the normal range for this patient. Due to the few number of vertebral fractures in the sample, correlation and regression analyzes were not feasible and so the study was not able to evaluate whether vertebral fractures are associated with HCV infection.

## Discussion

Our results demonstrated that non-cirrhotic untreated CHC men have lower total hip and trochanter BMD when compared to healthy men. Only a few studies had evaluated BMD measurements in non-cirrhotic CHC patients and their results are conflicting due to methodological questions [13]–[15]. In our present sample we were able to demonstrate that HCV infection affects BMD at the proximal femur independently of liver function, current cirrhosis or anti-viral medication.

In spite of the lower BMD values in CHC patients when compared to healthy men, mean BMD at all skeletal sites were within normal range. It is interesting to note that both CHC patients and healthy controls exhibited high prevalence of overweight and that is well recognized as having a protective effect on bone density [27]–[30].

Total hip BMD is highly influenced by trochanteric BMD since the trochanter occupies the largest area of the proximal femur. The lower values for total hip BMD observed among CHC patients may be explained by the significantly lower BMD measurements at the trochanter in these subjects. In agreement with this finding, other authors have also observed a significant decrease in trochanter BMD in CHC patients, regardless of the liver function [14].

Reduced current physical activity associated with higher prevalence of prior prolonged immobilization were also observed among CHC patients as compared to healthy controls and that may also have influenced our results. Lower mechanical load could be related to the lower values for trochanteric BMD observed among CHC patients. Studies with hemiplegic patients [31] and cosmonauts [32] have demonstrated the association between reduced weight-bearing activities and lower femur BMD. The effect is probably due to an increase of the number of osteoclasts and a decrease in the osteoblast population [33].

Hepatic dysfunction with the intervenient vitamin D deficiency has been associated with low bone density. A direct correlation has been reported between the severity of liver fibrosis and low bone density in non-cirrhotic CHC patients [15]. The issue is however controversial and in CHC patients without cirrhosis the association with low BMD has not been confirmed in all studies. Hofmann et al [34] reported no correlation between non-cirrhotic hepatic disease and BMD measurements. They have also found no association between BMD measurements and disease duration, viral load and lifetime tobacco exposure. Our data have demonstrated no significant correlation between BMD measurements and the severity of liver inflammation and fibrosis at the biopsy, viral load, transmission model, estimated disease duration or alcohol intake. It is important to note that patients with alcoholism were excluded from our study.

Our results have also shown that non-cirrhotic CHC patients present no clinically relevant abnormalities in bone markers and calciotropic hormones, as previously observed [13]–[15], [34]. Mean serum 25OHD concentrations were considered sufficient in CHC patients, corroborating previous findings [14], [34]. To our knowledge this is the first study to classify non-cirrhotic CHC patients according to vitamin D status. No significant difference was observed in 25OHD status between CHC patients and healthy controls. That also suggests that liver injury in our patients was not important, at least in terms of vitamin D synthesis and hydroxylation.

The number of morphometric vertebral fracture was rather small in both groups precluding further analyzes. The absence of vertebral fractures may also result from the normal lumbar BMD values observed in both CHC patients and healthy controls.

Some limitations of the present study need to be pointed out. Our cross-sectional design prevented us of establishing a cause-effect relationship in our results as well as evaluating the long-term effect of HCV infection on bone metabolism and fracture risk. As observed earlier, the high prevalence of overweight in our population might also have influenced our results and could have positively impacted on BMD measurements in both groups. Results in women chronically infected with HCV might be somewhat different from those reported here for men.

This study is the first to evaluate BMD, bone metabolism and the prevalence of morphometric vertebral fracture in a significant number of men with CHC, without the classic confounding factors for low bone density (menopause, cirrhosis and antiviral therapy). Our results demonstrate that non-cirrhotic untreated CHC patients are at low risk for osteoporosis and fractures. We have also observed no significant abnormalities in bone and mineral metabolism and a vitamin D status similar to that seen for healthy men.
